# Research on Flesh Texture and Quality Traits of Kiwifruit (cv. Xuxiang) with Fluctuating Temperatures during Cold Storage

**DOI:** 10.3390/foods12213892

**Published:** 2023-10-24

**Authors:** Ranran Xu, Qian Chen, Yizhao Zhang, Jiali Li, Jiahua Zhou, Yunxiang Wang, Hong Chang, Fanxiang Meng, Baogang Wang

**Affiliations:** 1 Institute of Agri-Food Processing and Nutrition, Beijing Academy of Agriculture and Forestry Sciences, National R&D Center for Fruit Processing, Beijing 100093, China; xuranran@iapn.org.cn (R.X.); 18800162193@163.com (Y.Z.); zhoujiahua@yeah.net (J.Z.); yunxiangjkkl@126.com (Y.W.); changhongch2008@163.com (H.C.); mfx18731230819@163.com (F.M.); 2 Institute of Agricultural Resources and Regional Planning, Chinese Academy of Agricultural Sciences, Beijing 100081, China; chenqian940910@163.com (Q.C.); lijiali@cau.edu.cn (J.L.)

**Keywords:** temperature fluctuation, kiwifruits, cold storage, flesh texture, qualities

## Abstract

Kiwifruits are often exposed to various temperature fluctuations (TFs) during postharvest transportation and storage. To evaluate the effect of TFs on the qualities of kiwifruits during storage, kiwifruits were stored at 2 °C, 2 °C or 5 °C (TF2 °C–5 °C, alternating every 12 h), 2 °C or 7 °C (TF2 °C–7 °C, alternating every 12 h) for 3 d before long time storage at 2 °C. Observations revealed that kiwifruits stored at a constant 2 °C showed the lowest loss of weight and vitamin C because of minimized ethylene production and respiratory rate compared with that of TF2 °C–5 °C and TF2 °C–7 °C. Moreover, the results of RT-qPCR verified that the expression levels of genes encoding polygalacturonase, β-galacturonidase, and pectin methylesterase were significantly increased by the treatment of TF. Hence, TF accelerated the degradation of cell walls, softening, translucency, and relative conductivity of the flesh of kiwifruits. In addition, the impact of TF2 °C–7 °C on kiwifruits was more significant relative to TF2 °C–5 °C. The present study provides a theoretical basis for kiwifruit during cold storage.

## 1. Introduction

Kiwifruit *(Actinidia chinensis* Planch.) is one of the most highly-appreciated fruits by consumers worldwide owing to its delicious taste and nutritional value, including its high content of vitamin C. As a kind of typical climacteric fruit, kiwifruit has a short storage period [1]. There is an apparent physiological after-ripening process, and the fruit is easily infected by fungal pathogens during postharvest storage, resulting in deterioration of quality, loss of flavor, and even severe rot [2,3]. It is extremely perishable because of its susceptibility to senescence and postharvest disease, making it challenging to store, transport, and sell commercially [4,5].

Temperature is one of the most significant factors in maintaining the quality and extending the shelf life of fruits and vegetables [6]. Cold storage is an effective way to decay ripening and extend the shelf life after harvest by reducing respiration and oxidase activity [7,8]. Cold chain transport effectively maintains the color and texture of kiwifruit [9]. The storage of fruits and vegetables requires an optimal temperature with minimal fluctuation [10]. However, there are inevitable temperature fluctuations (TFs) during long-distance transportation, including improper operation, imprecise temperature control devices, and other problems. Current research on TF has focused on the dynamics of quality loss in frozen foods during storage [11,12,13], with less research related to the impact on the quality of fresh fruit and vegetables. Serious temperature fluctuations accelerated the quality degradation of table grapes in a cold chain quickly [14]. In addition, the quality of mushrooms, broccoli, and mature-green tomatoes, including flesh browning and a loss of firmness and weight, were significantly impacted by temperature changes, which were packaged in a modified atmosphere [15]. The decline in food quality caused by temperature changes is cumulative and irreversible [16,17].

Translucency is considered one of the symbolic signals of chilling injury symptoms due to fruit flesh texture deterioration during cold storage [18]. Liquid fills the intercellular gap in the translucent flesh area, resulting in a water-soaked appearance [19]. Transparency leads to a loss of food quality in terms of taste and flavor. Moreover, translucent fruits are more susceptible to physical damage and fungal diseases, which are detrimental to storage and transportation [19]. Integrated metabolome and transcriptome analyses show that translucency is a complex process involving genes belonging to multiple metabolic pathways and various metabolic processes [20]. In addition, transparency may be related to metabolic dysfunction from membrane damage [21] and the collapse of adjacent tissue [22]. Transparency is a frequent phenomenon in the flesh of kiwifruit during storage. However, to date, little has been reported previously on the transparency of kiwifruit after harvest.

The current temperature fluctuation range of cold chain land transportation is mostly 3~5 °C, and the transportation time is up to 3 d based on actual logistics transport data from southern to northern China. This is one of the major constraints faced by wholesalers and retailers. However, studies focused on the effect of TF on the postharvest physiology of kiwifruit are limited. Accordingly, this study simulated different temperature fluctuation patterns during transportation to monitor the physiological parameters affecting postharvest life and quality of kiwifruit during cold storage, including respiratory metabolism, flesh texture, ripening, edible quality, and related gene expression. The overall purpose of this research study was to study the effects of different temperature fluctuations during transportation on subsequent kiwifruit quality during cold storage.

## 2. Materials and Methods

### 2.1. Sample Collection and Treatments

**Figure 1:**
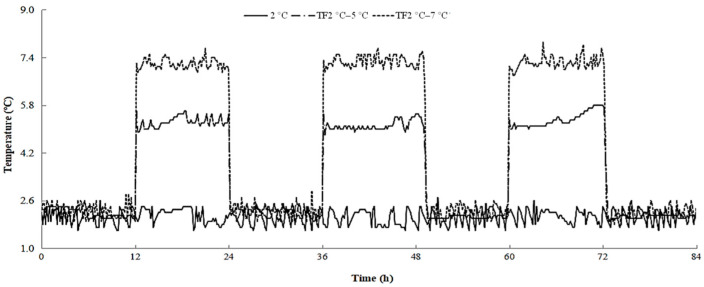
Temperature changes in kiwifruit packaging during storage. The control fruit were always stored at 2 °C during storage. TF2 °C–5 °C fruit were stored at 2 °C or 5 °C, alternating every 12 h. TF2 °C–7 °C fruit were stored at 2 °C or 7 °C, alternating every 12 h.

“Xu Xiang” kiwifruits were harvested at commercial maturity from a commercial orchard located in Guiyang district, Shaanxi, China. The kiwifruits were transported to the laboratory within 2 h and selected according to their uniformity of size and color. Those with visible mechanical injury or defects were discarded. The fruits were randomly divided into three groups, with 540 fruits in each group. One group was stored at 2 ± 0.5 °C, which served as a control. One group was stored at 2 °C or 5 °C, alternating every 12 h, served as TF2 °C–5 °C. Another group was stored at 2 °C or 7 °C, alternating every 12 h, served as TF2 °C–7 °C. All treatments were performed at 85–95% relative humidity (RH) (Figure 1). After 72 h, all fruits were transferred to storage at 2 °C and 85–95% RH. The pulp of fruits was combined, frozen in liquid nitrogen, and stored at −80 °C until assaying for gene expression.

### 2.2. Microscope Observations

The samples for optical microscope observations were only obtained 21 d after storage. The pulp was cut into small pieces (2–3 mm^2^) and fixed in formaldehyde–acetic acid fixative solution (50% ethanol: 5%formaldehyde: 5%acetic acid = 18:1:1) for 24 h. Then, the pulp was dehydrated through step-wise increases in ethanol concentration (50, 60, 70, 80,90, 95 and 100%). The pulp sections were then treated with carbon dioxide to the critical drying point using a critical point drier (HCP-2; Hitachi, Tokyo, Japan). The dried pulp sections were glued to a metal table with double-sided adhesive to expose the observed parts. The samples were then coated with gold using an ion sputtering meter (E1010; Hitachi) and observed using an optical microscope (MODEL ECLIPSE Ci-S; Nikon, Tokyo, Japan) [23].

### 2.3. Translucency Assessment and Decay Incidence

**Figure 2:**
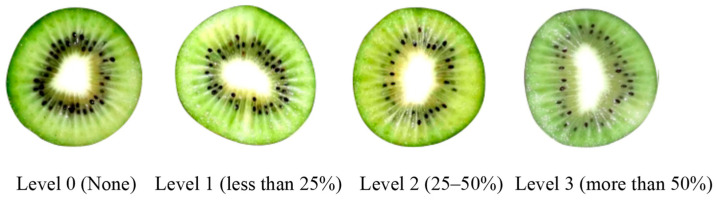
Grading of translucency in kiwifruit pulp.

The translucency incidence of the fruit pulp was visually evaluated and scored based on the percentage of the total cross-sectional area containing the translucency number, using the following grading scale: 0, no symptoms; 1, less than 25%; 2, 25–50%; 3, more than 50%, as shown in Figure 2 [24]. The translucency index was expressed as:

(1) $$\mathrm{Translucency}\;\mathrm{index}=\frac{\sum [\mathrm{Translucency}\;\mathrm{level}\times \mathrm{number}\;\mathrm{of}\;\mathrm{fruit}\;\mathrm{at}\;\mathrm{this}\;\mathrm{level}]}{\mathrm{Total}\;\mathrm{number}\;\mathrm{of}\;\mathrm{fruit}}$$

Kiwifruits from different treatment groups were collected separately every week. The total number of kiwifruit and rotten kiwifruit (rotten spot diameter ≥ 2 mm) in each treatment were counted, and the decay incidence was calculated according to the following formula:

(2) $$\mathrm{Decay}\;\mathrm{incidence}=\frac{\mathrm{Number}\;\mathrm{of}\;\mathrm{rotten}\;\mathrm{fruit}}{\mathrm{Number}\;\mathrm{of}\;\mathrm{total}\;\mathrm{fruit}}\times 100\%$$

### 2.4. Respiration Rate and Ethylene Production

The respiration rate and ethylene production were measured in 30 fruits (containing 3 replicates) every week using a portable gas analyzer (F-950; FELIX, Washington, DC, USA) [25]. The ethylene production and respiration rate were presented as μL kg^−1^ h^−1^ and mL kg^−1^ h^−1^, respectively.

### 2.5. Fruit Quality Assessments

Firmness was measured using a handle penetrometer (FT327; FACCHINI SRL, Alfonsine, Italy) equipped with an 8 mm diameter tip. Total soluble solids (TSS) were measured using a pocket refractometer (PAL-1; ATAGO, Tokyo, Japan). Titratable acidity (TA) was determined from freshly homogenized juice samples from ten fruits per replicate using an auto titrator (809 Titrando; Metrohm, Herisau, Switzerland). The content of vitamin C was determined by high-performance liquid chromatography (HPLC) according to the methods of Gai and Wang [26]. Weight loss was measured by weighing each fruit at harvest and each subsequent assessment day. Cumulative weight loss was expressed as a percentage value determined by deducting the initial weights from the final weights, dividing by the initial weights, and multiplying by 100 percent (%).

### 2.6. Relative Conductivity Determination

The relative conductivity was measured by a conductivity meter (FE38; METTLER TOLEDO, Shanghai, China) [27]. Discs of 2 mm from kiwifruit flesh were punched out using a punch with an inner diameter of 1 cm for relative conductivity determination. The discs were placed in a large beaker, and then 20 mL of ultrapure water was added. The water was poured off after shaking for 10 min. Then, samples were rinsed three times with ultrapure water to remove excess pectin. The discs were placed in ultrapure water, and the electrical conductivity was measured before and after 20 min of boiling. The percentage of the ratio of these two values was the relative conductivity.

### 2.7. Reverse Transcription and Quantitative Real-Time PCR (RT-qPCR)

The RNA was extracted from fruit flesh tissue using an RNA prep Pure Plant Plus kit (Tiangen, China) according to the manufacturer’s protocol. Complementary DNA (cDNA) was synthesized by reverse transcription using the Invitrogen Super-Script™ III First-Strand Synthesis System (Carlsbad, CA, USA). RT-qPCR was performed using a 96-well plate in a Light Cycler1480II real-time PCR system (Roche, Basel, Switzerland) with an Ultra SYBR mixture (CW Bio Co., Beijing, China). The amplification of *Pbactin* sequence was used as an endogenous reference to normalize all data. The 2^−ΔΔCt^ method [28] was employed to determine gene expression. The primers are listed in Table S1. All analyses contained three technical replicates.

### 2.8. Statistical Analysis

All tests were repeated three times, and each repeated test served as a block in the statistical design. SPSS19.0 statistical software (SPSS Inc., Chicago, IL, USA) was used to perform the analysis of variance, followed by the least significant difference (LSD) mean comparison test.

## 3. Results and Discussion

### 3.1. Effect of Temperature Fluctuation on Micromorphology of Pulp Cell of Kiwifruit during Storage

**Figure 3:**
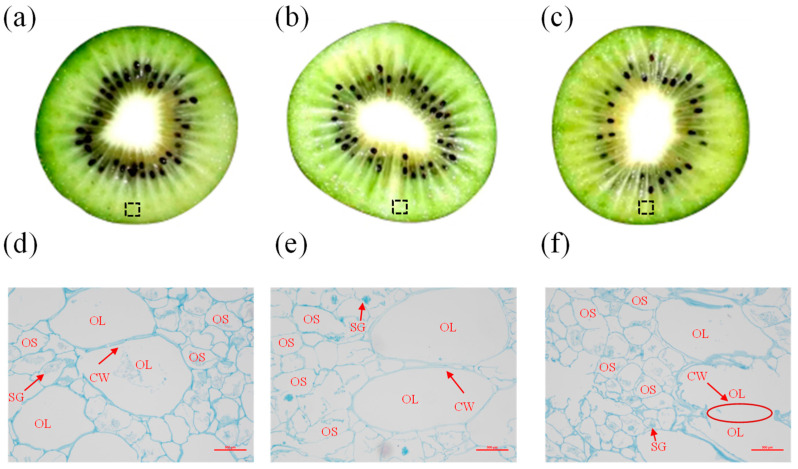
The fruit ((**a**): control, (**b**): TF2 °C–5 °C, (**c**): TF2 °C–7 °C) stored for 21 d were collected for micromorphology observation. The dotted line box in the fruit cross-section diagram is the current observation sampling position. Micromorphology of pulp cell was observed using an optical microscope ((**d**–**f**), 40× magnification, bar = 500 µm) in kiwifruit stored for 21 d. OL means large cell, OS means small cell, CW means cell wall, and SG means starch granules.

The effect of temperature fluctuation on the flesh tissue of kiwifruit was observed. The flesh of fruits in the TF2 °C–5 °C group (Figure 3b) and TF2 °C–7 °C group (Figure 3c) turned transparent and water-soaked significantly, compared with the control (Figure 3a). As the temperature fluctuation deepened, the degree of transparency became more acute. Changes in the microstructure of the cell wall of kiwifruit treated with different temperature fluctuations were observed using optical microscopy. As shown in Figure 3d, the exocarp cells of “Xu Xiang” kiwifruit were composed of thin-walled cells of both sizes, which were regularly round or oval in shape. The exocarp cells of kiwifruits stored at 2 °C for 7 d were regularly shaped and tightly arranged with the cell wall and starch granules intact. However, the starch granules of kiwifruits in TF2 °C–5 °C started to gel. The cell walls gradually loosened, and the boundaries between cells became blurred (Figure 3e). The cell damage of kiwifruits in TF2 °C–7 °C was intensified (Figure 3f). The cell walls of kiwifruit began to degrade and disappear. In addition, the whole tissue was filled with starch matrix. From the microscopic observation results, the transparency may be related to the degradation of the cell wall, which further leads to the penetration of the cell solution. The degradation of the cell membrane can indeed be attributed to various factors, including attacks from reactive oxygen species (ROS) [19]. Furthermore, the oxidative stress caused by ROS can lead to a disruption in lipid molecules in the cell membrane, ultimately compromising its integrity. Then, more liquid is released from cells and accumulates in the intercellular space of the translucent flesh. The disorders in water content and solutes, such as sugar in the intercellular spaces, may lead to transparency [19]. Degradation of the cell wall could be responsible for kiwifruit softening. The degradation of the cell wall resulted in the breakdown and degradation of polysaccharide molecules within it, such as pectin, cellulose, and hemicellulose [29]. Pectin serves as a crucial component in the middle lamella of the cell wall, and a decline in the cohesion of the cellular network may be the primary reason for the softening of many fruits [30]. Studies have shown that a reduction in the adhesion between cells caused by the dissolution of the middle lamella and the loss of cell divisions as well as cell turgor was accelerated under 5 ± 5 °C TF at 30 d of the storage, while constant temperature under 5 ± 0 °C maintained the integrity of the cell wall structure and firmness of apples [31]. Accordingly, temperature fluctuations promoted the degradation of cell walls and the rupture of starch granules, resulting in the transparency and softening of kiwifruits.

### 3.2. Effect of Temperature Fluctuation on Fruit Texture of Kiwifruit during Storage

**Figure 4:**
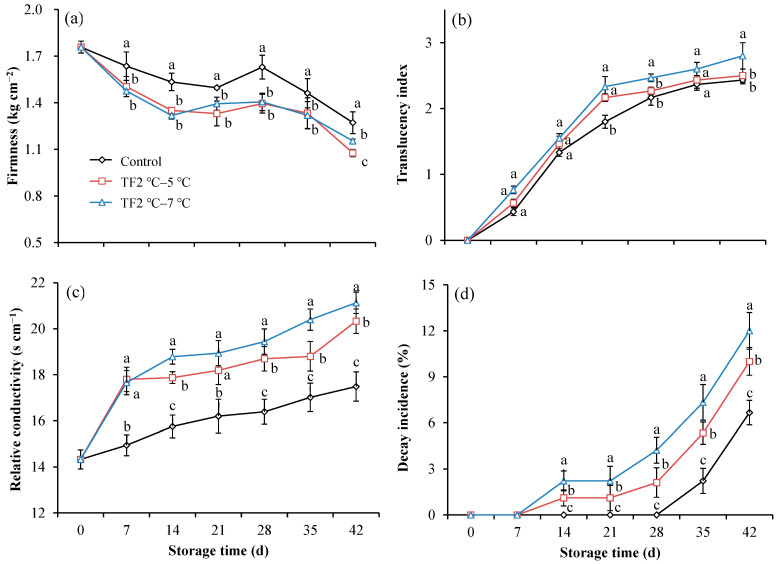
The firmness (**a**), translucency index (**b**), relative conductivity (**c**), and decay increase (**d**) of kiwifruit during storage. Each value is the mean for three replicates. The vertical bar indicates the standard deviation. Values marked with different lowercase letters (a–c) within the same time were significantly (*p* < 0.05) different according to the LSD test.

Firmness is one of the deteriorating factors that limit the postharvest quality of fruits. Hence, firmness is essential for the overall acceptance of fruit. The firmness of kiwifruit in the control group decreased from 1.76 kg cm^−2^ to 1.27 kg cm^−2^ after storage for 42 d (Figure 4a). The firmness of kiwifruits with temperature fluctuation was consistently lower than that of control during the storage period. At the end of the storage period, the firmness of the kiwifruits was decreased by the treatment of TF2 °C–5 °C and TF2 °C–7 °C compared with control by 15% and 9%, respectively. As the degree of temperature fluctuation increased, the degradation in firmness of peaches accelerated as the structural compounds such as pectin and hemicelluloses were degraded in the cell wall [32], which is consistent with our results. In addition, the disappearance of starch granules, the expansion of intercellular space, and the depolymerization of pectin and hemicellulose cause a decrease in kiwifruit firmness [33]. While the cellulose content and structure remained mostly similar, the length of the pectin polymer and the number of branches were reduced [33].

The translucency index of fruits treated with TF2 °C–5 °C and TF2 °C–7 °C significantly increased compared with the control group (Figure 4b). In particular, the translucency index was increased by 20% and 29% in the TF2 °C–5 °C and TF2 °C–7 °C treated fruit compared with that of control on 21 d, respectively. Translucency is closely related to the release of liquid from cells because of damage to the cell membrane. Fruit relative conductivity is often regarded as an important indicator of fruit cell membrane structure and integrity. The relative conductivity of control and treated fruits continued to increase during storage, although the control fruits always had the lowest relative conductivity (Figure 4c). As the temperature fluctuation aggravated, the degree of relative conductivity increased. Compared with the control, the relative conductivity was increased by 16% and 21% in the TF2 °C–5 °C and TF2 °C–7 °C treated fruit on 42 d, respectively. The increase in relative conductivity implied damage to the cell membrane. Multiple metabolic pathways are impacted once the membranes break down, such as ion leakage, finally leading to membrane rupture and cell death [34]. Therefore, it is essential to avoid temperature fluctuations to reduce membrane damage.

The decay incidence is one of the important indexes representing fruit storable capacity [35]. Kiwifruits in the treatment groups began to decay within 14 d, but did not occur in the control group until 35 days (Figure 4d). In addition, the decay incidence of the treatment groups was always significantly higher than that of the control throughout the entire storage period. The decay incidences of the TF2 °C–5 °C and TF2 °C–7 °C treatment groups were 50% and 80% higher than that of the control group on 42 d, respectively. The cell wall-mediated resistance is an important component of plant immunity. The damage to cell walls and cell membranes during TF made it easier for the fungus to invade fruit tissues, which exacerbated fruit rot [36].

The softening of various fruits and vegetables tends to accumulate during storage. Additionally, the loss of firmness can make them more susceptible to physical damage [31,37]. Moreover, an increase in membrane permeability of cells is associated with the translucency of the fruit flesh [38]. Temperature fluctuations during cold storage have been found to elevate the translucency index and relative conductivity of kiwifruit, a pattern consistent with the impact of temperature fluctuations on sweet cherries [39]. Consequently, the decay incidence was significantly exacerbated by temperature fluctuations (TF). Therefore, it is advisable to minimize TF treatments during storage.

### 3.3. Effect of Temperature Fluctuations on Respiratory Rate, Ethylene Production, and Quality of Kiwifruit during Storage

**Figure 5:**
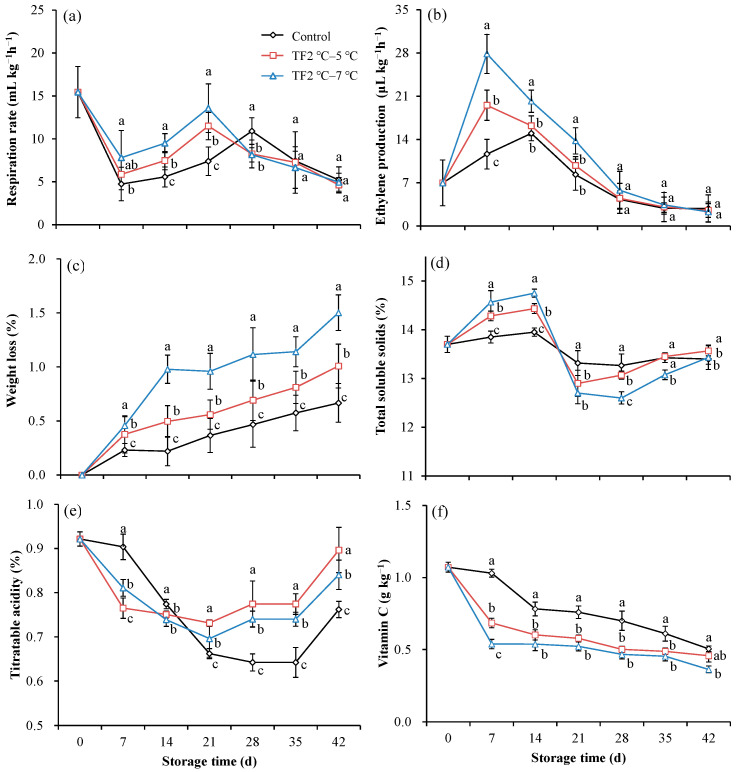
The respiration rate (**a**) and ethylene production (**b**), weight loss (**c**), total soluble solids (**d**), titratable acidity (**e**), and vitamin C (**f**) of kiwifruits during storage. Each value is the mean for three replicates. The vertical bar indicates the standard deviation. Values marked with different lowercase letters (a–c) within the same time were significantly (*p* < 0.05) different according to the LSD test.

As a kind of typical climacteric fruit, kiwifruit has both an obvious respiratory peak and an ethylene peak during storage. The respiratory rate of control kiwifruits rapidly decreased throughout the first 7 d of storage and then gradually increased over the next 21 d (Figure 5a). Subsequently, the respiratory rate decreased, eventually falling below the harvest rate. The respiratory rate of the fruit in the TF2 °C–5 °C and TF2 °C–7 °C groups was consistent with that of the control group. However, the respiratory peaks of the fruit in TF2 °C–5 °C and TF2 °C–7 °C groups appeared at 21 d of storage, 7 d earlier than that of the control group. The highest peak respiratory values were found in the TF2 °C–7 °C group, followed by the TF2 °C–5 °C group, and were lowest in the control group. The temperature fluctuation not only promoted earlier peak respiration but also increased peak respiration values, which is not conducive to maintaining fruit quality. Similarly, the temperature fluctuations promoted an earlier peak in ethylene by 7 d and increased peak ethylene values (Figure 5b). The ethylene peaks of the fruit in the TF2 °C–5 °C and TF2 °C–7 °C groups appeared at 7 d of storage, which were 30% and 86% higher than that of control, respectively. For postharvest products, the respiration rate and ethylene release rate reflect the process of fruit ripening and senescence [40]. Kiwifruits are thought to be particularly sensitive to low ethylene concentrations (i.e., 0.005~0.01 μL L^−1^) and may show excessive softening, resulting in fruit losses. Hence, avoiding substantial ethylene production is important for effective regulation of fruit quality [41]. In addition, the enhanced respiration promoted the loss of fruit quality losses, resulting in economic losses. TF accelerated the ethylene and respiration peaks in kiwifruit; at the same time, the occurrence of ethylene and respiration peaks in tomato [42], “Fuji” and “Golden Delicious” apples [31] under 5 °C was accelerated by TFs. Therefore, avoiding temperature fluctuation is crucial to maintaining suitable respiration and ethylene production.

Temperature fluctuation aggravated the weight loss of kiwifruits during storage (Figure 5c). The weight loss rate was dramatically higher in the TF2 °C–5 °C and TF2 °C–7 °C treated fruit than in the control by 51% and 125% on 42 d. The water loss was mainly due to the transpiration and respiration of the fruit itself. The water content of fruits is regarded as a predominant indicator because it directly relates to taste and physiological metabolic capacity [43].

The main metabolites that affect how kiwifruits taste are sugars and acidic chemicals, and their ratio is frequently employed as a measure of both flavor and maturity [44]. The temperature fluctuation led to a higher peak value and lower peak value of TSS content (Figure 5d). During the early stage of storage, a significant increase in the SSC of kiwifruits treated with TF may be due to more vigorous metabolic activities promoting the conversion of fruit starch and other non-sugars to soluble sugars, as well as a loss of water. Then, a subsequent significant decrease in the SSC of fruits treated with TF may be the result of increased SSC consumption by respiration. The TA content of the control decreased after storage (Figure 5e). The treatment with TF2 °C–5 °C and TF2 °C–7 °C increased the TA content of the kiwifruits by 18% and 10% at 42 d, respectively. The SSC/TA ratio, a fruit taste indicator, was significantly lower in TF-treated kiwifruits than in the control group at later storage periods because of the higher TA and lower SSC content, which led to a worse-tasting kiwifruit.

The most noticeable nutritional characteristic of kiwifruit is its high vitamin C content. Vitamin C, often referred to as L-ascorbic acid, is one of the most well-known antioxidant compounds found in kiwifruit, which is most strongly correlated with the total antioxidant activity of kiwifruit [45]. The vitamin C content gradually reduced in control fruit during storage, which was promoted by temperature fluctuations (Figure 5f). In particular, the vitamin C content of fruits treated with TF2 °C–5 °C and TF2 °C–7 °C decreased by 33% and 48% at 7 d, respectively. A possible explanation for the lower vitamin C content may be attributable to the higher enzyme activity in the oxidation of ascorbic acid caused by TFs [45].

Active respiration and high ethylene release cause the weight loss of fruit; furthermore, water loss is the predominant factor in mass loss. TFs not only promoted respiration and ethylene release but also significantly facilitated weight loss in kiwifruit. The increase in total soluble solids upon TF treatment during the first 14 d of storage may be due to the loss of water. The trend in TSS content in kiwifruits was the same as that in tomatoes [42]. In contrast, a reduction in vitamin C content was promoted by the TF, which was opposite to that in tomatoes [42]. The rapid reduction in vitamin C may be its use as a substrate in the rapid metabolism stimulated by TFs. Overall, a stable temperature is conducive to the maintenance of good fruit quality.

### 3.4. Effect of Temperature Fluctuation on Gene Expression of Kiwifruit during Storage

**Figure 6:**
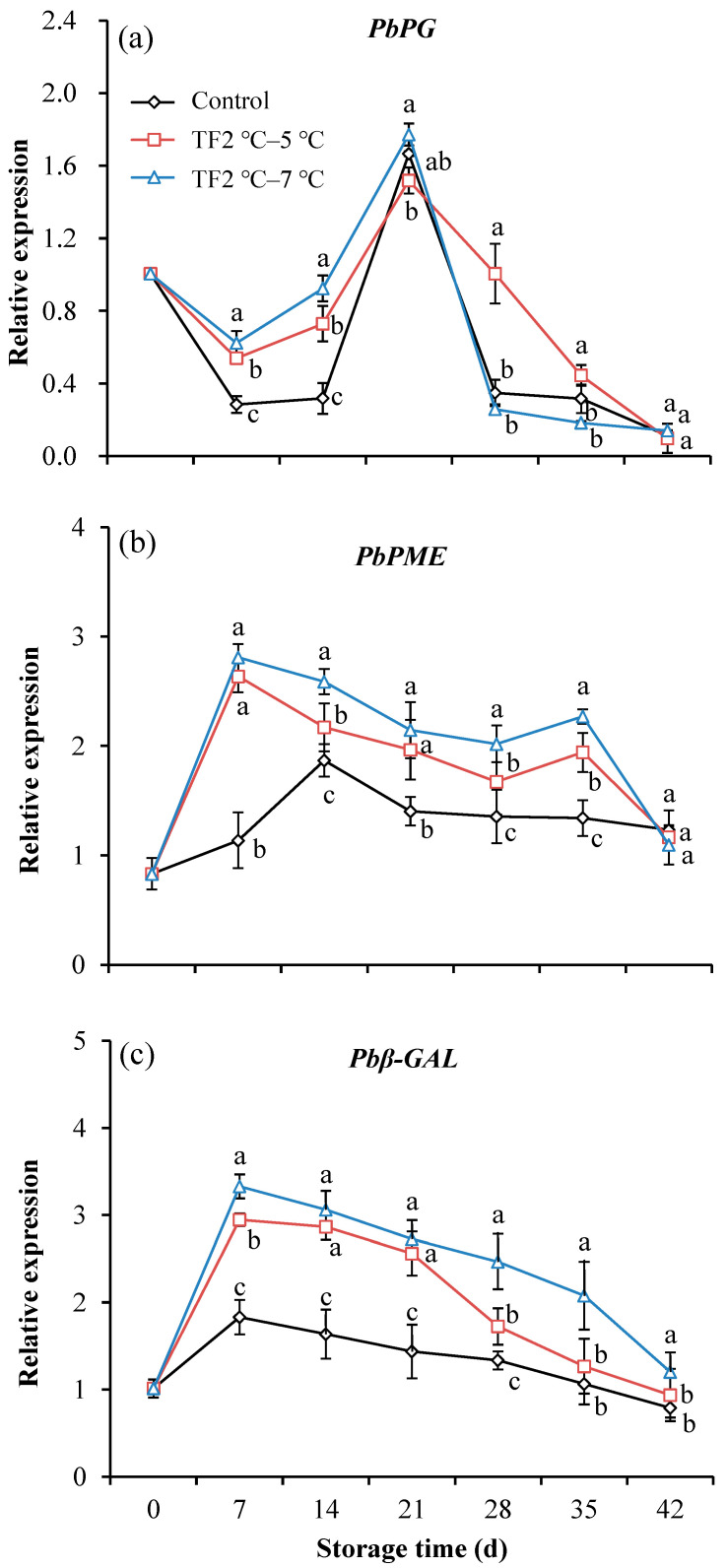
Genes expression of *PbPG* (**a**), *PbPME* (**b**), and *Pbβ-GAL* (**c**) of kiwifruit during storage. Each value is the mean for three replicates. The vertical bar indicates the standard deviation. Values marked with different lowercase letters (a–c) within the same time were significantly (*p* < 0.05) different according to the LSD test.

The cell wall metabolism of fruit is intimately tied to postharvest textural changes. Hemicellulose depolymerization, pectin solubilization and depolymerization, and neutral sugar loss from pectin side chains are examples of extensive cell wall alterations that change the mechanical properties of the components of the cell wall [46]. Pectin degradation is brought on by the constant conversion of acid-soluble pectin (ASP) into water-soluble pectin (WSP), which is carried out by the enzymes polygalacturonase (PG), pectin methylesterase (PME), and β-galactosidase (β-Gal) [47]. During the early softening stage of kiwifruit, it has been noted that a loss of galactans in homogalacturonan (HG), which is controlled by PG, results in a reduction in the integrity of intercellular connections [46]. This is followed by a loss of arabinose side chains in pectin and pectolysis. Additionally, the galactose residues in galactan are broken down by β-Gal [48]. The PME-modified HG demethylation mechanism may be crucial in controlling the rate at which kiwifruit softens [46]. To investigate the factors influencing softening, changes in the expression levels of genes encoding these enzymes were measured by RT-qPCR. In the control group, relative expression of *PbPG* decreased during the first 7 days, then rose to a peak on 21 d, and then declined again until the end of storage (Figure 6a). The treatment of TF2 °C–7 °C promoted the expression of *PbPG* in the early stages of storage. The relative expression of *PbPE* in control fruits peaked at 14 d and then gradually decreased (Figure 6b), while TF led to earlier peaks and increased peak values. The relative expression of *PbPE* in TF2 °C–5 °C and TF2 °C–7 °C fruits mainly higher than that of control fruits during storage. The relative expression of *Pbβ-Gal* in control kiwifruits slowly increased during the first 7 d of storage and then decreased (Figure 6c). The relative expression of *Pbβ-Gal* in the TF2 °C–5 °C and TF2 °C–7 °C groups was 1.6-fold higher and 1.8-fold higher than that in the control group at 7 d, respectively. One of the fundamental causes of softening is the structural development of pectin in the primary wall and intercellular layer of fruit under the influence of enzymes associated with cell wall metabolism, including PG, PME, and β-Gal [49,50]. TF accelerated the expression of *PG*, *PME*, and *β-Gal*, which further promoted the softening of kiwifruit.

## 4. Conclusions

The effect of temperature fluctuation of varying degrees during transportation on subsequent kiwifruit quality during cold storage was studied. Compared with temperature fluctuations at 3 °C (TF2 °C–5 °C) and 5 °C (TF2 °C–7 °C), kiwifruits stored at a constant 2 °C maintained better quality and flesh texture status during subsequent storage. Temperature fluctuations significantly promoted the production of ethylene and CO_2_ compared with no temperature fluctuation, which accelerated the loss of weight and vitamin C. Furthermore, the expression levels of *PbPG*, *PbPME,* and *Pbβ-GAL* were up-regulated by the TF treatment compared with the control. Hence, the softening, translucency, and relative conductivity of kiwifruits were aggravated. In addition, temperature fluctuation promoted a decrease in SSC and an increase in TA after storage, leading to a decrease in fruit flavor. Fruit storage quality degraded when temperature fluctuation became more severe. Therefore, in order to maintain better postharvest quality and prolong storage of kiwifruits, TFs should be avoided as much as possible. The results of this study provide a theoretical basis to elucidate the mechanism underlying the effect of temperature fluctuations on kiwifruit quality during the subsequent storage period.

## Data Availability

The data that support the findings of this study are available on request from the corresponding author upon reasonable request.

## Supplementary Materials

The following supporting information can be downloaded at https://www.mdpi.com/article/10.3390/foods12213892/s1, Table S1: Primer sequences used for RT-qPCR.
